# Confidence at the Slit Lamp: The Case for Accessible, Low-Cost Training

**DOI:** 10.7759/cureus.100103

**Published:** 2025-12-26

**Authors:** Surinder S Dosanjh, Shruti Chandra, Rahila Zakir

**Affiliations:** 1 Ophthalmology, Imperial College Healthcare NHS Trust, London, GBR; 2 Faculty of Medicine, Imperial College London, London, GBR

**Keywords:** education, ophthalmology, simulation in medical education, skill training, slit-lamp

## Abstract

Effective use of the slit lamp is a fundamental skill for ophthalmologists. However, ophthalmology teaching plays a diminishing role in many medical school curricula, with limited or no slit-lamp training. As a result, many residents begin specialty training without formal prior experience.

A 90-minute introductory course was delivered to first-year ophthalmology residents across the deanery. The session comprised four short talks covering slit-lamp components, corneal examination, posterior segment examination, and gonioscopy with Goldmann applanation tonometry. Three of the four talks were delivered by second-year ophthalmology residents, with fourth-year ophthalmology residents providing senior oversight and addressing more complex queries. This was followed by a practical session in which residents practiced gonioscopy, direct and indirect fundoscopy, and corneal examination using SimulEYE and Reti Eye models. Pre- and post-course feedback was collected using a 7-point Likert scale.

Nine residents attended the session, and eight completed post-course feedback. All reported no prior formal ophthalmology experience. Mean confidence scores improved across all domains: slit-lamp knowledge (2.9→5.1), corneal and anterior chamber examination (3.0→4.75), posterior segment examination (1.9→4.5), indirect ophthalmoscopy (1.3→3.9), and intraocular pressure measurement (2.0→4.38). All respondents found the session helpful in building their confidence with basic slit-lamp skills and recommended that it be included in the ST1 educational programme.

The course significantly improved confidence in core slit-lamp and examination skills. Our model was non-paid, centralised, and resource sustainable, using reusable equipment and a peer-to-near-peer teaching structure with senior oversight. This format promoted open dialogue, reduced hierarchy, and improved engagement. Given its accessibility and the positive feedback received, we support its incorporation into trainee educational programmes and its replication across other regions.

## Introduction

Competent use of the slit lamp is an essential part of ophthalmic practice and forms a core element of the UK Ophthalmic Specialist Training (OST) curriculum set by the Royal College of Ophthalmologists [1]. Effective use of the slit lamp is key to performing clinical assessments and informing clinical decision-making. Despite this, many trainees start specialist training with minimal or no prior experience using the equipment. This is largely attributable to the gradual reduction of ophthalmology teaching within undergraduate medical programmes in the United Kingdom, where opportunities to develop eye-related clinical skills have steadily declined over time [2,3].

Consequently, residents starting their first year of ophthalmology training often begin with limited background knowledge and little practical familiarity with slit-lamp examination. This early gap can undermine confidence, slow the development of examination skills, and lead to inconsistent training experiences in the initial stages. Increasing service demands within the NHS further exacerbate these challenges, as opportunities for informal, workplace-based teaching can vary widely between units [4]. One approach to addressing this is the implementation of structured, centralised teaching [5].

Peer-to-near-peer teaching provides one potential solution. Learning from colleagues who are only slightly more advanced in training can be more relatable for new trainees. This type of teaching often encourages more open discussion, reduces perceived hierarchy, and creates a learning environment where new trainees feel comfortable asking questions and discussing difficulties. To support early training needs, we developed a low-cost, centrally organised slit-lamp teaching course specifically aimed at residents in their first year of ophthalmology training. Holding the course around one month into the training year allowed participants enough clinical exposure to identify their own challenges, which they could then address during the session. In this study, we describe how the course was designed and delivered and evaluate its impact on improving confidence in using the slit lamp safely and effectively.

## Materials and methods

The course was conducted in a single day, lasting approximately one and a half hours, and took place about one month into the first year of training. Nine residents participated, supported by a teaching team of six: two fourth-year ophthalmology residents, three second-year residents, and an Education and Simulation fellow. The fellow was responsible for organising, managing, and facilitating the session. The course was scheduled on a weekend, during a period of reduced clinical activity. With approval from the local directorate and senior nursing team, permission was granted to use the outpatient department, where no patient services were taking place. This allowed access to multiple slit lamps within the clinic. Equipment used for the course included standard fundoscopy lenses (90D, 78D, and SuperField), a gonioscopy lens, and a 20D lens. A binocular indirect ophthalmoscope was borrowed from the local ophthalmology department for teaching purposes.

The session opened with a brief introductory teaching segment lasting approximately 30 minutes. During this time, residents attended four focused presentations covering the following topics: an overview of slit-lamp components, corneal examination techniques, methods for assessing the posterior segment, and the principles of gonioscopy, along with Goldmann applanation tonometry. The presentations were delivered in the dedicated lecture space using digital slides. Residents were encouraged to ask questions, with a brief discussion period allocated after each talk. Most presentations were given by the junior ophthalmology residents, while the senior residents were present to supervise and address more complex questions. Talks focused on the principles of examination, with expansion on areas relevant to the topic. For example, the talk on assessment of the anterior chamber angle encompassed examination technique, evaluation of angle structures, Shaffer grading, and the use of Goldmann applanation tonometry, alongside broader clinical interpretation. This teaching was further consolidated during the subsequent practical session.

Following the talks, the remainder of the session was devoted to hands-on skills development. Delegates were navigated to the outpatient space, where no clinical activity was scheduled. Multiple slit lamps were available to facilitate the practical component of the session. Over the course of approximately one hour, residents moved between several stations where they had the opportunity to practise anterior and posterior segment examination using a slit lamp, perform indirect ophthalmoscopy, and practice the practical steps involved in intraocular pressure measurement using both a rebound tonometer and a Goldmann applanation tonometer. Stations were arranged in a circuit, and within each station, residents were provided individualised feedback by faculty. During the practical component, we made use of simulation models already present within the department, such as Reti Eyes and SimuEYE models supplied by Altomed [6,7]. 

**Figure 1:**
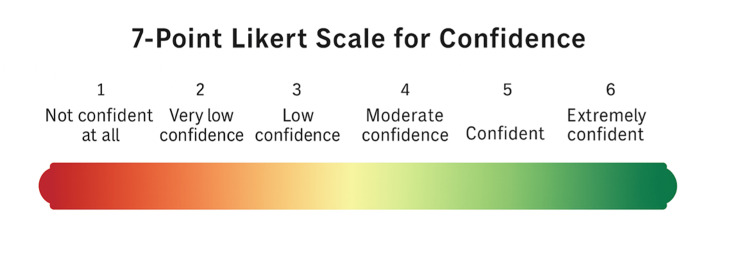
7-point Likert scale used to assess trainee confidence before and after the course This figure shows a schematic created to depict the Likert scale used. The scale ranged from 1 = not confident at all to 7 = extremely confident, with intermediate descriptors indicating very low (2), low (3), moderate (4), confident (5), and very confident (6) levels. This was adapted from the original 5-point scale [10]

The Reti Eye models were used in two configurations: either attached to the slit lamp via the clamp provided or laid flat on a desktop. This enabled fundoscopy practice at the slit lamp and further practice in the use of an indirect ophthalmoscope. The SimulEYE SLT model was useful in demonstrating good practice when examining angle structures [8]. To facilitate the use of the models, we used the SimulEYE slit lamp stand, which was also available within the department [9]. Participants completed anonymous pre- and post-session questionnaires. Confidence was rated using an adapted 7-point Likert scale. This is an adaptation of the original Likert scale, which consists of five response options [10]. The 7-point format was adopted to enable greater sensitivity and discrimination between responses [11]. The scale is shown in Figure 1.

## Results

A total of nine residents attended the course. Of these, seven completed the pre-course questionnaire, and eight completed the post-course questionnaire. Before the session, residents were asked about any previous clinical experience in ophthalmology, including foundation placements, clinical fellow posts, or other formal ophthalmic roles. All seven respondents reported having no prior clinical experience in ophthalmology.

**Figure 2:**
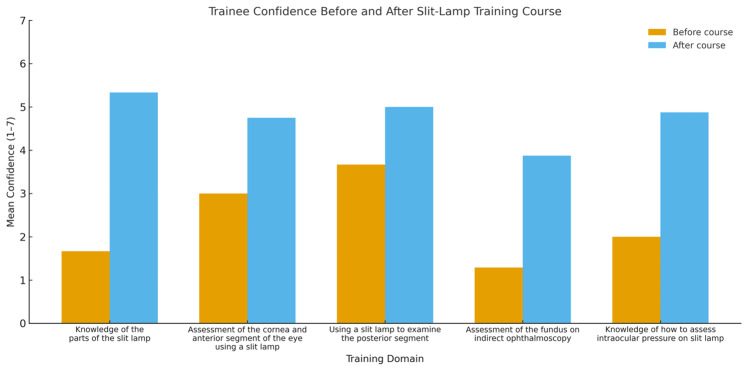
Ophthalmology resident confidence ratings The figure shows mean confidence ratings of participating residents across five clinical domains before and after the slit-lamp training course, assessed using a 7-point Likert scale adapted from the original 5-point scale [10]

Across all five assessed domains, residents demonstrated notable improvements in confidence following the course. Figure 2 provides a graphical summary of these changes. Confidence in identifying the parts of the slit lamp increased from a mean score of 2.85 to 5.13, reflecting a shift from very low/low confidence towards confident. Confidence in assessing the cornea and anterior segment improved from 3.0 to 4.75, corresponding to a transition from low confidence to a level between moderate and confident. Confidence in examining the posterior segment using the slit lamp also increased substantially, rising from 1.86 (not confident at all/very low confidence) to 4.5 (moderate confidence/confident). For indirect ophthalmoscopy, confidence improved from 1.29 to 3.88, indicating movement from not confident/very low confidence more towards moderate confidence. Finally, confidence in measuring intraocular pressure in the clinic increased from 2.0 to 4.38, representing an improvement from very low confidence to a level between moderate confidence and confident.

In the post-course questionnaire, additional questions were employed to explore attitudes towards the session. All eight respondents reported that the session was useful in helping them become more comfortable with slit-lamp use. Furthermore, all participants agreed that similar sessions should be incorporated into the first-year training programme in the future. All eight trainees also indicated that introducing a fee for this type of course could present a financial barrier to access.

Tutor performance was also evaluated. All six tutors received ratings, and all respondents reported that each tutor created an environment where questions were welcomed, supporting open discussion and learner engagement.

## Discussion

Ophthalmology has gradually been receiving less emphasis in many UK medical school programmes, with slit-lamp examination forming a minimal part of these programmes [2,3]. Often, many residents begin ophthalmology training with minimal practical experience in the use of a slit-lamp. This trend was reflected by those who attended the course, none of whom had previously worked in an ophthalmology post before starting their first year of training. This course offered a structured, centralised, low-cost method of tackling this knowledge gap. The confidence data collected showed that the session had a positive impact across all the domains assessed. Residents reported feeling more assured about their ability to use the slit-lamp and related examination techniques. Although confidence in indirect ophthalmoscopy improved, it remained lower than the other skills assessed. To address this in future iterations, we aim to increase the time allotted to gaining practical experience in indirect ophthalmoscopy.

One of the key elements of the course was the use of peer-to-near-peer teaching. Residents taught by colleagues only slightly more advanced in their training often find the learning experience more relatable [12]. Those in near-peer roles have recent experience of the same learning curve and can anticipate common difficulties, which may help to reduce anxiety. This dynamic appeared to be effective in our session, as all respondents commented that they felt tutors were open to questions. This structure also benefits the tutors themselves by giving them opportunities to strengthen their own understanding while developing their teaching skills. Any limitations related to the tutors’ level of expertise were mitigated by having senior trainees available to provide oversight and answer more advanced queries.

The course was also designed to move beyond traditional lecture-based learning. While short lectures were included to introduce key concepts, a large portion of the teaching relied on discussion and hands-on interaction. Educational theory suggests that learners engage more confidently when they feel secure, supported, and part of an inclusive environment, as described in motivational frameworks such as Maslow’s work on human needs [13]. Encouraging residents to openly question and discuss their early clinical challenges enabled deeper learning than lectures alone, in line with Freire’s emphasis on dialogue-based education with a movement away from purely didactic forms of education [14]. The involvement of near-peer tutors complemented this style by creating a warm and welcoming atmosphere that invited questions and discussion. Some argue that the use of near peers enables social congruence, allowing the learners to feel more at ease [15].

The course prioritised environmental and financial sustainability, aligning with the NHS-wide goal of improving environmental sustainability [16]. Reusable materials such as slit-lamp models were used, greatly reducing the use of single-use items and keeping the environmental footprint low [17]. The course was cost-free to run, as the host unit already owned artificial eyes and stands, which were one-off purchases and can be reused for future iterations of the course and for other training programmes. In future iterations, we hope to acquire a more reliable and accurate model for the assessment of intraocular pressure. However, the cost-to-benefit ratio will be considered as we wish for this course to remain free for residents to attend. All participants stated that introducing a fee may establish a barrier to access, echoing broader concerns about the rising financial burden of medical training [18]. While paid courses certainly have a great deal of value where material or staffing costs are high, increasing the number of accessible, low-cost, centralised teaching opportunities may help reduce disparities in early training experiences.

Those who attended were unanimous in their view that the course was worthwhile and should become a regular part of the teaching programme. Holding the session approximately a month after residents started their posts appeared beneficial, as it allowed them to experience challenges in real clinical settings and attend with specific questions. The high tutor-to-delegate ratio facilitated personalised instruction, effective troubleshooting of practical issues, and targeted feedback.

## Conclusions

This course offered a practical and affordable way to build confidence in the use of the slit lamp among new ophthalmology residents who often begin training with limited prior exposure. Near-peer involvement fostered the creation of a friendly and open learning environment and helped tutors to consolidate their own skills, whilst maintaining oversight by more senior residents. The use of existing equipment and reusable materials kept the course cost-free and accessible. Delivering the session a month into training enabled residents to identify areas of difficulty that could be addressed during the session. Repeating the course annually and adopting similar low-cost models more broadly may support more consistent and equitable early training experiences.
